# Financial risk protection at the bedside: How Ethiopian physicians try to minimize out-of-pocket health expenditures

**DOI:** 10.1371/journal.pone.0212129

**Published:** 2019-02-12

**Authors:** Ingrid Miljeteig, Frehiwot Berhane Defaye, Paul Wakim, Dawit Neema Desalegn, Yemane Berhane, Ole Frithjof Norheim, Marion Danis

**Affiliations:** 1 Research Group in Global Health Priorities, Department of Global Public Health and Primary Care, University of Bergen, Bergen, Norway; 2 Department of Research and Development, Helse Bergen Health Trust, Bergen, Norway; 3 Centre for Medical Ethics and Priority Setting, College of Health Sciences, Addis Ababa University, Addis Ababa, Ethiopia; 4 Biostatistics and Clinical Epidemiology Service, National Institutes of Health, Bethesda, Maryland, United States of America; 5 Addis Continental Institute of Public Health, Addis Ababa, Ethiopia; 6 Department of Bioethics, National Institutes of Health, Bethesda, Maryland, United States of America; RTI International, UNITED STATES

## Abstract

**Background:**

Out-of-pocket health expenditures can pose major financial risks, create access-barriers and drive patients and families into poverty. Little is known about physicians’ role in financial protection of patients and families at the bedside in low-income settings and how they perceive their roles and duties when treating patients in a health care system requiring high out-of-pocket costs.

**Objective:**

Assess physicians’ concerns regarding financial welfare of patients and their families and analyze physicians’ experiences in reducing catastrophic health expenditures for patients in Ethiopia.

**Method:**

A national survey was conducted among physicians at 49 public hospitals in six regions in Ethiopia. Descriptive statistics were used.

**Results:**

Totally 587 physicians responded (response rate 91%) and 565 filled the inclusion criteria. Health care costs driving people into financial crisis and poverty were witnessed by 82% of respondants, and 88% reported that costs for the patient are important when deciding to use or not use an intervention. Several strategies to save costs for patients were used: 37–79% of physicians were doing this daily or weekly through limiting prescription of drugs, limiting radiologic studies, ultrasound and lab tests, providing second best treatments, and avoiding admission or initiating early discharge. Overall, 75% of the physicians reported that ongoing and future costs to patients influenced their decisions to a greater extent than concerns for preserving hospital resources.

**Conclusion:**

In Ethiopia, a low-income country aiming to move towards universal health coverage, physicians view themselves as both stewards of public resources, patient advocates and financial protectors of patients and their families. Their high concern for family welfare should be acknowledged and the economic and ethical implications of this practice must be further explored.

## Background

Out-of-pocket (OOP) expenditures for health care pose barriers to accessing health services and impose great financial risk on populations in low-income settings. Studies show that even small OOP costs can drive patients and families into poverty [1]. There is no general agreement on the definition of when health expenditures become catastrophic. A commonly used definition is household OOP expenditure in excess of 40% of household income (after covering the cost of basic needs)[2]. The definition might be problematic to use in low-income-countries (LIC) as most people are poor and unable to meet their basic needs. An alternative approach has been to look at payments as catastrophic based upon the health and economic implications the payments have at household level, e.g. when families have to borrow or sell their belongings to deal with the costs[3, 4]. Health expenditures can be related to use of private clinics or informal or traditional providers, with variation between countries and in different regions (urban/rural)[2]. Concerns about OOP expenditures have been studied in the US and show that even in high-income countries with widely available health insurance, patients may suffer substantial financial burdens due to OOP expenditures[5]. The burden is greatest for poor patients and for patients who have chronic and life-threatening illnesses. In many LIC, public health care is mostly free of charge, but additional costs for travel, medicines, diagnostic tests, food and shelter for accompanying persons can force the patient or family to pay substantial amounts when public health services are used. In addition, family income might decrease, due to illness-related reduction in the number of working individuals in the household.

Ethiopia is a particularly interesting country to study as it is the second-most populous country in Africa and it currently has a special focus on reducing OOP expenditures[6]. Ethiopia has great geographic, socio-economic, cultural and religious diversity. About 80% of the population lives in rural areas. Recent improvements have been reported for many health and development indicators[7], and key indicators relevant to understanding health development in Ethiopia are shown in Table 1. Ethiopia has a three-tier health service system. At the first level (district level), the health system comprises of primary hospitals (with population coverage of 60,000–100,000 people), health centers (1/15,000–25,000 population) and their satellite health posts (1/3,000–5,000 population) that are connected to each other by a referral system. Then the two next levels comprises of regional and referral hospitals. Ethiopia is scaling up essential health care packages through health extension workers, while at the same time implementing advanced medical treatments such as renal transplants, cardiac surgery and cancer treatment in selected public hospitals in the larger cities[8, 9].

**Table 1:** Selected health, development and poverty indicators of ethiopia[7, 10,11].

	
Life expectancy at birth (years)	65
Total fertility rate	4.1
Infant mortality rate (per 1000 live births)	50
Stunting in children under 5 years of age (%)	40.1
Population below poverty line (Poverty headcount ratio at $1.90 a day (2011 PPP) (% of population)	30
Hospital to population ratio (2013)	1:564173
Number of hospitals (by levels/types) (2013)	125
Physicians (GPs and Specialist) to population ratio (2013)	1:32132
Total number of general practitioners (2013)	1213
Total number of Specialists (2013)	331
Health expenditure as % of GDP	4.7
Per capital total expenditure on health (US$)	20.77
Out of pocket payments (as % of total health expenditure)	34

The newest household health utilization and expenditure survey from Ethiopia showed that *government facilities provided 75% and 78% of the total outpatient and inpatient services respectively*, *and that 50% of the total OOP was paid to government hospitals and health centers(11)*. *The rest of the OOP was for private-for-profit (47%) and non-profit organizations*. *Of the total OOP spending*, *45% were used for drugs and medical supplies*, *16% were used for diagnosis and investigation*, *12% covered food and accommodation expenditures (including for those that are accompanying the patient) and 10% was used on transport*. *While 4% was used on inpatient treatment*, *96% was used on out-patient treatment*. *Most physicians working in public hospitals in Ethiopia see both inpatients and outpatients in their daily clinical work*.

Households use various sources like savings, borrowing, using loans or mortgages, and selling assets or livestock to meet OOP expenditures [12, 13]. One study from Ethiopia showed that typical coping mechanisms for families in rural areas were reducing food consumption (19%), asset sales including food stock (30%) and borrowing (19%), while as many as 21% did not have any coping mechanism at all[14].

Increased knowledge about determinants and health consequences of OOP expenditures has led many low-income countries, including Ethiopia, to develop public health strategies and health financing reforms such as community-based health insurance or social health insurance to reduce OOP expenditures and provide financial risk protection (FRP)[15]. Also, opinion leaders and donors have argued that avoiding catastrophic health expenditures has an important role on the fair path to universal health coverage [16]. The discussions have so far concentrated on macro-level recommendations and priorities. The role of physicians and other health care workers in facilitating a fair path to universal health coverage has hardly been explored.

We have previously documented Ethiopian doctors’ experiences of resource constraints and bedside rationing[17]. We found that physicians encounter numerous dilemmas due to resource scarcity, and they report lack of adequate guidance for how to handle them. The consequences for patients and professionals are substantial. As many as 59% of the respondents had daily or weekly regrets about their choice of profession due to resource constraints. While there are diverging opinion on the role physicians should have in minimizing costs and act as gatekeepers of resources, studies from both high-income and low-income countries have shown that health workers try to ration scarce resources in order to save costs for the institution[18–21]. But it is unclear how much they focus on patient and family financial concerns when they limit the use of potentially beneficial health care treatment. Except for a few qualitative studies from LICs showing how doctors and nurses experience a sense of responsibility for the whole family’s welfare when initiating treatment that imposes relatively high costs on a poor family[22], we have not been able to find any studies on health care providers’ concerns about the financial risks faced by patients and their approach to protecting against such risks.

The objective of this study was to assess physicians’ concerns regarding the financial welfare of their patients and their experiences in reducing catastrophic health expenditures for patients in Ethiopia. We compare their inclination to provide financial protection to patients with their willingness and approaches to limiting expenditure of institutional health care resources.

## Method

### Study design, participants and setting

The analysis reported here is based on a nation-wide, cross-sectional survey of physicians working in public hospitals in Ethiopia, including specialists, general practitioners (GPs) and residents in various specialties with more than one year of clinical experience. We have previously reported from the same survey on physicians’ experiences with resource scarcity and priority setting dilemmas. Extensive descriptions of the survey methods, the development and cognitive testing of the questionnaire and the data collection can be found there[17].

### Sampling procedure

Ethiopia is divided into nine regions that are characterized as being urban, rural or pastoralist, and two city administrations. We randomly selected two urban, two rural and two pastoralist regions for study inclusion. Most of the specialists work in Addis Ababa city administration, therefore Addis Ababa was also purposively included. Multistage sampling was conducted and weighting was done according to numbers of hospitals in each region. In all, 49 hospitals were included; at each of them all physicians (specialists, residents and GPs) working at the time of the study were invited to participate in the survey.

### The questionnaire

In this study we present results from several questions in the survey. Among them were questions on physicians’ concerns regarding the financial welfare of their patients and their reported practices of reducing catastrophic out-of-pocket health expenditures for patients and their families as well as the institution they worked in. The list of potential strategies for saving costs for the institutions was developed with an initial list similar to a list presented in Samia et als study among European GPs and internalists (21). The list was discussed among a broad range of physicians (both specialists and residents, 30 in total) attending an ethics training course, and was revised accordingly. The list was then extended to strategies for saving costs for the patient and family. The lists were further revised after pilot testing the whole questionnaire, as previously described (17). Two separate questions and lists were presented for the participant–one concerning institutional costs and one concerning patient and family costs.

### Data collection

Physicians were recruited in their department at the end of their morning meetings or at their work place in the period July—November 2013. One of the authors (FBD) visited all the participating hospitals and distributed the self-administered questionnaire.

### Statistical analysis

Data were coded, entered using EPI INFO. Data were analyzed using descriptive statistics and McNemar’s test to compare strategies used to save costs for the institution and for the patient and family.

### Ethical considerations

The research was conducted in accordance with the principles for medical research as described by the Helsinki Declaration. There were no known risks for the participants, and they did not directly benefit from participation in this study. All participants gave written consent. Data were handled and analyzed anonymously. Ethical approval was obtained from the IRBs of Addis Ababa University College of Health Sciences and the US National Institute of Health, and exempted by the Norwegian Regional Committee for Medical Research Ethics.

## Results

### Respondents

Of the 640 distributed questionnaires, 587 responded (response rate 91%). Physicians with less than one year of service were excluded and final analysis was done on 565 respondents. Respondents’ characteristics can be seen in Table 2.

**Table 2:** Respondents characteristics. All respondents were government employed. Analysis done on valid N, excluding missing and not applicable.

		Number who answered this question
**Women/Men (%)**	21 /79	563
**Mean Age (Range)**	31	555
**Age group (%)**		555
< 31	68	
31–40	21	
41–50	9	
> 50	4	
**Undergraduate medical training Ethiopia (%)**	94	551
**Postgraduate medical training Ethiopia (%)**	94	278
**Mean service year.**	6	540
**Years in practice (%)**		540
1–5 years	70	
6–10 years	15	
11–20 years	9	
> = 21 years	8	
**Professional status (%)**		556
GPs	49	
Specialists	24	
Residents	27	
**Have private practice**	38	565
**Average work hour/week in government**	46	525
**Average work hour/week in private**	20	28
**Average number of patients/week**	135	525
	**Involvement in medical academics (%)**		72	413
**Involved as:**				
Instructor	53			
Resident	36			
Researcher	6			
Others	6			
**Involvement in planning and decision-making at the hospital (%)**	28	559

### Clinician perceptions of patient out-of-pocket health expenditures and potential consequences for the families

**Fig 1:**
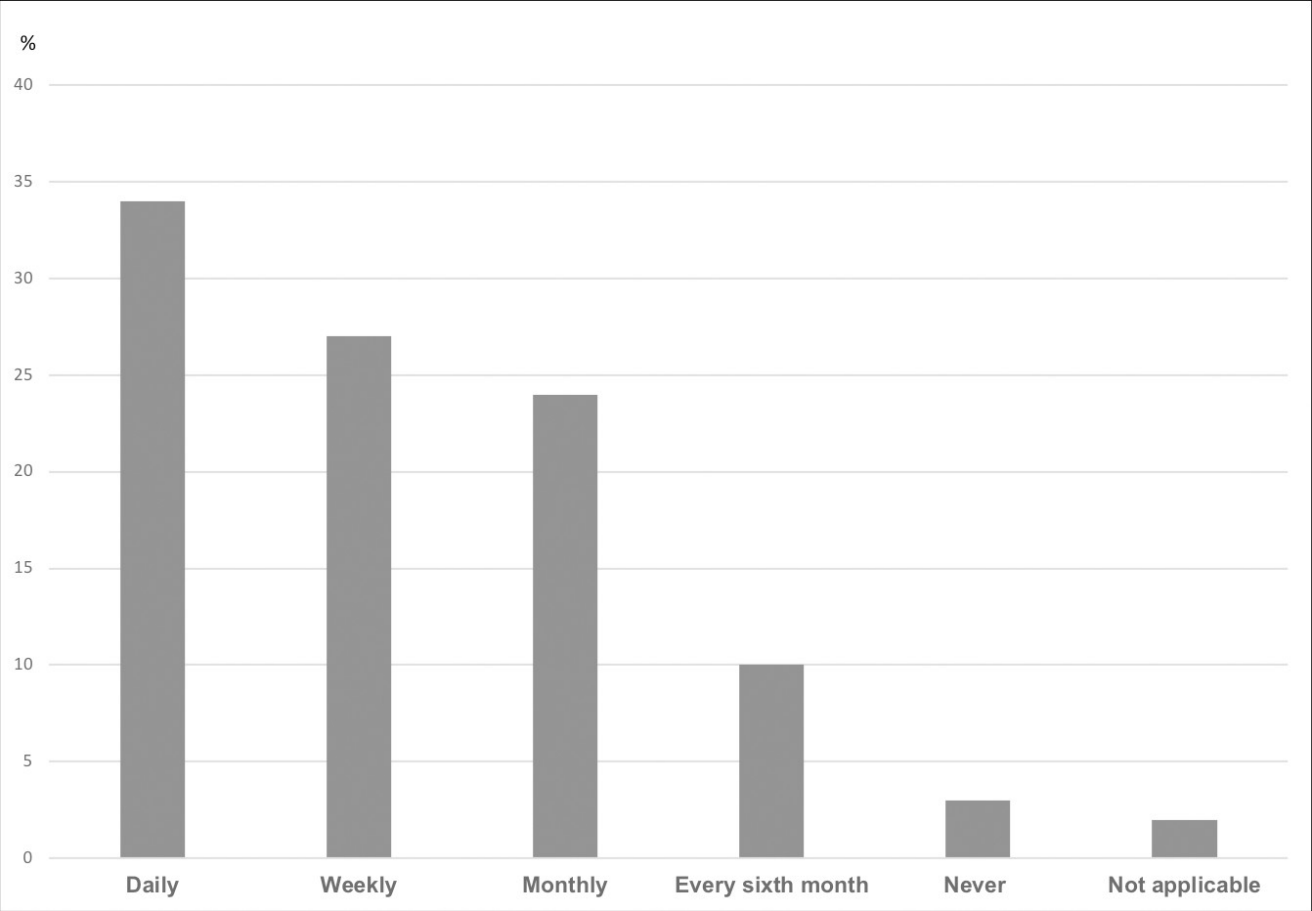
Physicians reported experiences of encountering patients who have problems that cannot be treated because they cannot afford the treatment (N = 550).

Among our respondents, 97% encountered patients who had problems that could not be treated because they could not personally afford treatment (Fig 1).

Of the respondents, 82% strongly or partly agreed that they had seen health care expenditures drive people into financial crisis (See Table 3).

**Table 3:** Physicians’ perception on their patients’ health expenses, their own roles and duties and their reported practices of protecting their patients and their families from health care costs. *For some of the criteria the total do not sum up to 100% due to rounding.

	Strongly agree (%)*	Partly agree (%)	Neutral (%)	Partly disagree (%)	Strongly disagree (%)	Number who answered this question
**Physicians´ perceptions regarding patient out-of-pocket expenditures, financial risk and resource scarcity**						
In my setting, there is lack of enough resources to provide standard medical care	79	17	1	1	1	536
I have seen that health care costs drive people into financial crises	49	33	10	4	3	530
I find that the patients in the private clinics are often forced to pay for diagnostics/treatment they will not benefit much from	34	35	18	6	7	529
I see examples of patients that are not well informed about the total treatment costs in the private clinics	37	38	18	4	4	528
I see examples of patients that are not well informed about the total treatment costs in the public health care system	29	46	14	6	3	530
**Physicians reported attitudes regarding their roles and duties**						
Physicians have the obligation to protect the health care system from avoidable expenses	67	25	6	1	1	533
Physicians should adhere to cost effective standard interventions instead of more expensive interventions that has small proven advantages over the standard intervention	64	25	6	4	1	531
Physicians should try to protect poor families from out-of-pocket health expenses, by recommending cheaper, but second best treatment	34	39	15	7	5	533
Denying medically beneficial but costly services to patients interferes with the doctor-patient relationship	29	35	16	9	12	523
**Physicians reported practices**						
Costs for the patient is important for me when I decide to use or use an intervention or not	62	26	7	3	2	530
I try to act as my patients advocate to make sure they get the medical services they need	53	31	10	4	1	525
The financial burden on the health care system is important when I decide to use an intervention or not	37	37	18	6	3	530
Ongoing and future costs to the patient influence my decisions more than use of hospital resources	33	41	17	4	2	524
If I see that the patient is poor, I do not let the patient know about the expensive options	11	26	16	21	26	529

Our informants were all working in public hospitals, and 96% strongly or partly agreed there is lack of enough resources to provide standard medical care. Also, 68% of them strongly or partly agreed that patients are often forced to pay for diagnostics or treatment that they will not benefit much from in the private clinics, and 75% saw examples of patients who were not well informed about total treatment costs in the private clinics or in the public health care system.

### Clinician attitudes and practices

The majority of the physicians (88%) reported that costs for the patient are important when deciding to use or not use an intervention (Table 3). They reported being obliged both to protect the patients and their families from costs and trying to function as their advocates to ensure they get the medical service they need. They felt obliged to protect against out-of-pocket expenses by recommending cheaper, but second-best treatment (73%). Providing an explanation of costs and benefits of a treatment alternative for the patient or family and giving recommendations on affordable options was done daily or weekly by 70% of them (numbers not shown in table). Table 3 also shows that many found that denying medically beneficial, but costly services to patients, interferes with the doctor-patient relationship.

Simultaneously, 74% of informants were concerned about the costs for their institutions. They agreed that physicians are obliged to protect the health care system from avoidable expenses (92%), and should adhere to cost effective standard interventions instead of more expensive interventions that has small proven advantages over the standard intervention. In total, 74% agreed to some degree (34% strongly agreed and 41% partly agreed), while 6% partly or strongly disagreed with the statement: “Ongoing and future costs to the patient more often influence my decisions than use of hospital resources”.

### Strategies used to limit out-of-pocket expenditures and provide financial risk protection at the bedside

**Fig 2:**
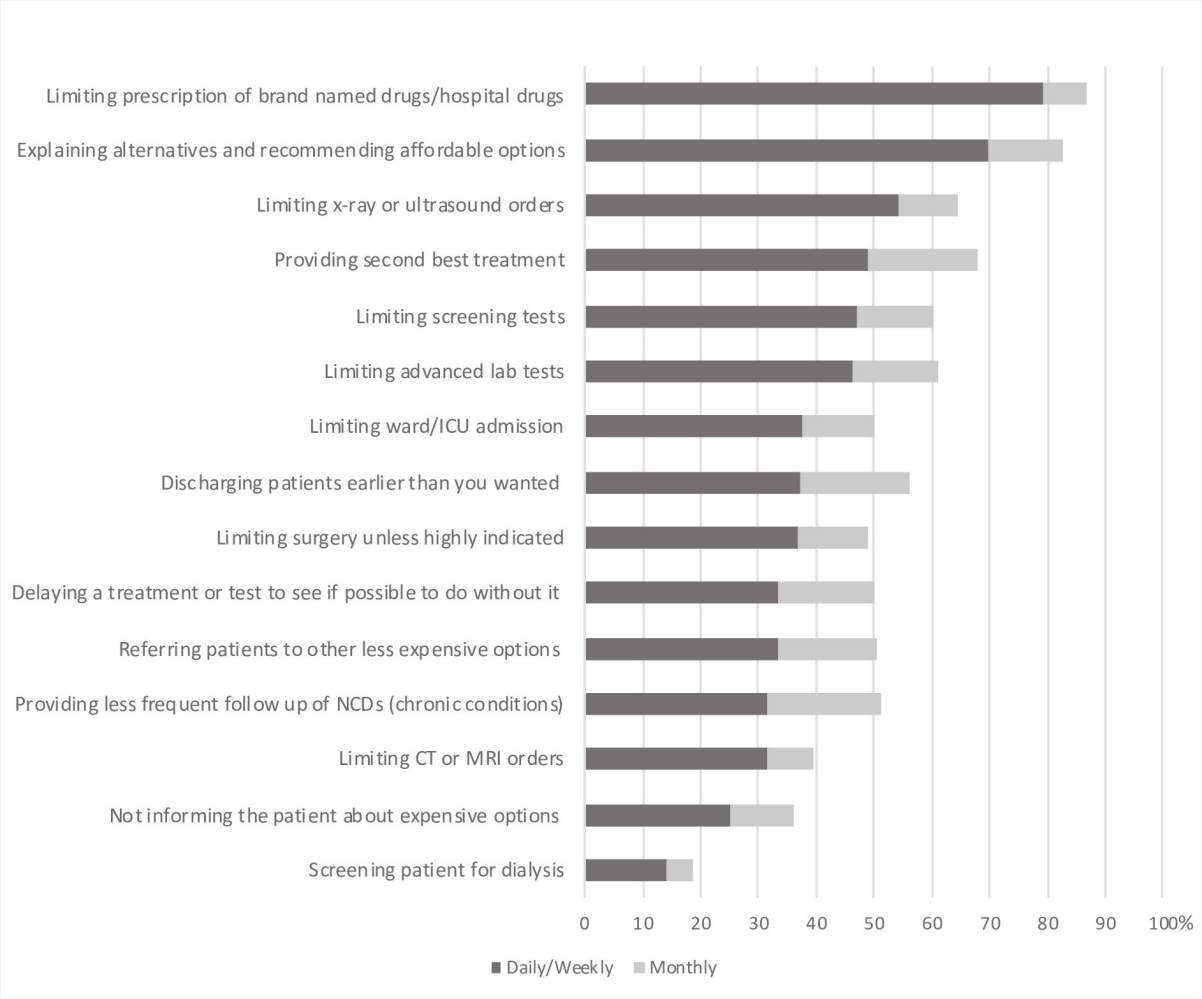
Strategies Used by Physicians to reduce costs for the patient and family. Shown by descending order of daily/weekly use.

Twelve of the fourteen strategies listed in our questionnaire were used daily or weekly by more than 30% of the respondents. The five most commonly reported strategies to save costs for the patient or family, with between 46–79% using the strategy daily or weekly, were limiting the prescription of brand name drugs, limiting x-ray or ultrasound studies, providing second best treatment, and limiting screening test or advanced lab tests (Fig 2).

### Comparing strategies used to restrict expenses for the families or for the institutions

When comparing the strategies used to restrict expenses covered by the institution or the family, we found that, overall, physicians more often protected families from expenses than the institution, as shown in Table 4. Only the strategy “Not informing patients about options that would lead to high costs for them” was not reported significantly more often in order to protect patients and families compared to protect the institution. The ranking of strategies, sorted by how often they were used, is almost the same. Limiting drugs, investigations, providing second best treatment and not admitting or discharging early were also the most common strategies used to restrict expenses for the institution.

**Table 4:** Comparison of the strategies used daily or weekly by the physicians to save costs for the patient and family and the institution they work in. *not significant p-value **Strategies asked only for either saving costs for the patient and family or for the institution.

Strategies used daily or weekly to save costs	For the patient or family. (%)	For the institution (%)	Significance level (P-value)
Limiting prescription of brand named drugs/hospital drugs	79	42	<0.0001
Explaining alternatives and recommending affordable options**	70		
Limiting x-ray and ultasound orders	54	39	<0.0001
Prioviding second best treatment	49	34	<0.0001
Limiting screening tests*	47		
Limiting advanced lab tests	46	30	<0.0001
Limiting ward/ICU admission	38	32	0.036
Discharging patients earlier than you wanted	37	26	<0.0001
Limiting surgery unless highly indicated	37	27	<0.0001
Delaying a treatment or test to see if possible to do without it	34	23	<0.0001
Referring patients to other less expensive options	33	28	0.069*
Prioviding less frequent follow up of NCDs (chronic conditions)	32	18	
Limiting CT or MRI orders	32	15	<0.0001
Not informing the patient about expensive options	25	22	0.28*
Screening patient for dialysis	14	9	0.0003
Refuse expensive drug requested by patients or families**		21	

## Discussion

We found that the majority of Ethiopian physicians working in public hospitals encounter patients who are at great financial risk even when they seek care in government funded institutions. When encountering these patients, physicians hesitate to provide otherwise recommended and potentially beneficial medical treatments either to protect them from further expenditures or because they know that they could not pay. Many of the physicians reported that they try to compensate for this by various strategies and second best options. Although respondents reported great concern for expenditures in the government-owned institution they work in, they put significantly more effort into protecting the patient and the family from burdensome health care expenses. Such findings have not been reported previously and deserve further discussion.

### Patient poverty and out-of-pocket health expenses are forcing doctors to make difficult trade-off between health and other sources of welfare

We found that our respondents work in a context where much treatment is unavailable in their hospital and/or is not provided for free even if it is within a public institution. The household health utilization and expenditure survey from 2015/2016 showed that 50% of the total OOP was used to get services in governmental institutions[11]. Studies show that families in Ethiopia experience high OOP expenditures even to get health care that is given high priority in national policies and that is available in public institutions. Memirie et al show how families in rural Ethiopia have high OOP to get outpatient and inpatient pneumonia treatment for their children in public health centers [23], while Tolla et als study show the high total costs of preventive treatment for families[24]. Onarheim et al show how families with a sick neonate in rural and semi-urban Ethiopia face small, but for them substantial costs, and some of them make the hard choice of not seeking care for their newborn in government institutions in order to protect the family from financial catastrophe [25]. These studies offer an important contribution to the literature by contradicting the view that OOP for health care primary are caused by patients seeking private services. Our study corresponds to these studies, as our informants were asked to respond on what they experience when working in the public hospital, and 82% have witnessed families being economically deprived after paying for medical care.

Doctors are trained through medical school and have taken the Hippocratic Oath to act in the best interest of their patients. Our respondents observe sources of suffering of patients and families in addition to disease and pain. And when choosing between what can be described as two evils, they seem to sometimes assess the health care expenditures as causing more significant harm than forgoing treatments. In our previously published paper on bedside rationing based on this same survey, we found that 94% claimed that scarcity of resources often or sometimes forced them to make a difficult choice[17]. Based on the results in this paper, we infer that one of these difficult choices is whether or not to provide well-known, efficient and available treatment for a patient immediately at hand, knowing that the trade-off is much poorer prognosis or even death for the patient versus, catastrophic economic consequences for the patient and family.

### Second best or nothing at all–adjustment strategies to protect the patient’s wallet

In our survey we listed 14 strategies we know are used, based on self-reports from doctors in Ethiopia (FDB, DND, OFN and participants from different specialities attending an ethics course) and through a planned piloting of the questionnaire. Still, we were quite surprised at the high frequency of use of all the strategies. Twelve of the fourteen strategies were reported to be used by one third of respondents every day or every week, and the five most common strategies were used among 46–79% on a daily or weekly basis. Recommending generic medicines is a policy stated strategy to reduce health care costs in Ethiopia[26], and our study confirms that the doctors follow this. Imaging studies using ultrasound and x-rays are still limited in government institutions, the services are quite expensive, and are sometimes only available to patients at private clinics. Strategies such as not screening for dialysis or limiting MR or CT-orders were not reported very frequently, but in our study we asked all kind of specialists and general practitioners working in various departments the same question. The majority of them are therefore unlikely to have to regularly make decisions regarding these services. Therefore we find that the use of the strategies of limiting MR or CT-orders is still high when adjusting for the expected need to do this among all specialists treating all kinds of patients.

Distribution of scarce resources at a clinical level can be conducted in several ways. In the literature six forms of bedside rationing are described: 1) denial of potential beneficial treatment 2) deflection, that is directing to other alternatives 3) deterrence, that is discouraging would-be beneficiaries from accessing services, 4) delay, 5) dilution, that is spread resources like providing suboptimal care and 6) termination, that is withdrawal of treatment[27]. Kapiriri et al. describes how doctors in Ugandan hospitals are using all these forms when deciding to use hospital resources or not on a patient [28]. This is shown in our study too, but what is not documented before is that they use the same strategies also to protect the patient and family from resource-use.

### Triple role of physicians in low-income context: patient’s advocate, gatekeeper of the institutions resources and the family’s financial protector

The majority of respondents viewed themselves as patients’ advocates, aiming to make sure that the patient gets what he/she needs. This finding is consonant with other studies showing the strong moral obligation and relational bond between patients and their doctors[29]. Previous studies have shown that physicians are torn between the often-conflicting roles of being patient advocates and gatekeepers of government or institutional resources[21, 30, 31]. The tendency to provide care to the identified person at hand is described as the rule of rescue[32]. This tendency leads health workers to try to do good for the immediate patient in front of them even if this inclination is not the most efficient use of resources[33]. Our survey findings seem to suggest that doctors often forgo treatment of the patient at hand not merely to save resources for others, but also because offering a treatment may not, on balance, be best for the patient herself or himself or the family because of competing welfare needs. Confronted with patients who cannot pay, knowing the consequences for families who must use their small savings, borrowing or other coping mechanisms, they choose to restrict beneficial treatment of the patient to protect the family from economic ruin and suffering. In studies of treatment limitation of premature neonates in India, Miljeteig et al found that the family’s economic situation and ability to take care of a child with special (i.e. more costly) needs were two of the major factors that doctors considered when deciding whether or not to withhold treatment[22]. Our study might seem to be an illustration of a paternalistic doctor-patient relationship[34]. But we did not directly ask our informants how they reached decisions to forgo providing treatment. Except from the finding that they do not always inform about the expensive options or referral possibilities, we do not know whether they are including patients or families in these decisions. A number of authors have recommended that clinicians talk to patients explicitly in the course of medical decision-making so that financial concerns can be taken into account despite reluctance or barriers to such conversations[35–38]. In focus groups conducted with patients in the US, members of the public are interested in hearing about the costs of potential treatments during the physician-patient encounter. They are more receptive to talking about their personal costs than the cost to society [39]. At the same time, some poor patients worry that they will automatically get second best treatment if the physician focuses on their financial concerns. The reality that there are choices to be made when weighing financial wellbeing and medical gains, argues for such explicit conversations during medical decision-making. Strategies for such conversations have been put forth[39]. Endorsement of such explicit discussion presumes that one has already endorsed shared decision-making between physicians and patients. Yet shared decision-making is not necessarily the norm in resource-poor settings where high patient volume leaves clinicians with very little time to talk to their patients. The results we found in this study should lead to more research on the feasibility and benefits of shared decision making with patients and their families in low-income settings, with a special focus on how to ommunicate about costs and the family´s ability to pay.

Our findings illustrate the extension of the physicians’ moral responsibility and role to include economic advice and implicit or explicit protection of costs or stewardship in contexts with high health care costs. The concept of stewardship has so far been used at a health policy level, introduced in the WHO report “Health systems: improving performance” from 2000 and has been discussed and debated after that[40]. Saltman et al examine the implications and potential benefits and challenges when the state aims to make decisions that are both normatively based and economically efficient[41]. Our study findings highlight the extent to which clinicians understand and can contribute to addressing the financial burdens that patients face in the course of receiving healthcare. Their perspective may be particularly helpful as decisions are made about benefit coverage on the path to fulfilling the universal health care provision. Since the stakes are so high, and the results of the decisions to forgo treatment for the sake of families is so significant for patients and families, attention to this issue and the resolution of physician´s decision maker roles as economic protectors or stewards should include public deliberation.

Until quite recently in countries such as Ethiopia, doctors have been few, they have had a very high status and the patients have both trusted and obeyed their health care provider[42]. Now, both the public and the health workers themselves are questioning the quality and the accountability of the health care system[43]. Working in a setting where they cannot trust that the patients get what they need, might increase the perceived obligation to safeguard the patient or family more than saving costs for the institution.

### Is it ethically permissible for physicians to trade their patient’s potential health benefit against the family’s welfare?

Our informants use different strategies to withhold information or forgo health services to the patients to protect them and their families from expenditures. Their concern for the family financial wellbeing was quite overwhelming. Is this ethically permissible? Our results indicate that clinicians struggle to achieve a net benefit for their patients. In so doing they weigh the underlying principles of beneficence and non-maleficence and often prioritize protection of the patient and/or their family from expenses. The challenge lies in that in the priority setting between health benefit for the patient and the welfare benefit for the family, there are potential harms and burdens and this needs to be explicitly discussed. In this setting, the consequences for the patients who do not get proper health care services are profound and sometimes fatal. Also, the burden of disease in the area might be masked by this practice; how can the government know the magnitude of unmet health needs and high out of pocket expenses? It is hard to openly discuss value-laden trade-offs face-to-face with a sick patient and family, but at the policy level there should be more deliberation about the trade-offs. There is general agreement on the importance of transparency of reasons and involvement of stakeholders in priority decisions at a policy level. In many low-income countries the implementation of universal health coverage (UHC) and essential health care packages are discussed and planed. UHC is defined by the World Health organization as “all people receiving quality health services that meet their needs without being exposed to financial hardship in paying for the services”[16]. Our study reveals the importance of in-depth studies of real life health costs and bedside decision-making as well as the need for value deliberations and clearer guidance for clinical decision-makers to make sure that decisions taken are ethically reasonable.

### Implications for clinical practice and policy

Physicians have an important role as decision makers and a great responsibility for distribution at the micro level, especially when there are extremely limited resources and no explicitly articulated national priorities or guidelines available for how to distribute and deal with catastrophic health expenditures. They are the contact point for patients in the health care system and are likely to best understand and appreciate the impact on financial ramification of the underinsured. Their voice in public discussions concerning health and financial protection should be strengthen and there is a need to prepare them with more skills and resources to make ethically justifiable decisions. But perhaps more importantly, our findings suggest the need to reduce out-of-pocket expenditures to a minimum for the most cost-effective, essential interventions so that poor patients can have some financial protection while receiving interventions that are most likely to offer benefit. While our physician survey did not focus on the other sources of financial burden for sick patients and their families, efforts to reduce the financial burden will require consideration of such programs as disability insurance and financial support for family caregivers.

### Strengths and limitations

This study provides new and highly relevant information regarding physician practices aimed at concrete, every-day financial risk protection of poor patients in Ethiopia. Our study includes all categories of physicians (approximately 38% of all physicians in Ethiopia[10]) working at all levels of the 49 hospitals in six randomly selected regions out of 11 in the country, and our response rate was particularly high. Our findings should be representative for the whole country, and we believe our findings are interesting for other countries and health systems in similar settings.

Our study also had limitations, and conclusions should therefore be drawn with some caution. The data on strategies used and priorities they make are self-reported and could be biased by the respondents presumed thoughts of what is expected of them. Doctors are aware that they are expected to care for their patients, to use resources efficiently and to collaborate. However, we do not believe expectations are clear when it comes to prioritizing the needs of patients versus others or how to protect the family from economic disaster. Another limitation of our study is that it did not include other health workers like nurses and medical officers who are working even more closely with families and know more about the consequences for the family welfare when a family member gets sick. This will be the focus in our second round of data collection. Our aim was to describe the current unexplored area of how providers perceive their roles and obligations of financial risk protection in public institutions. Our study should be extended by similar studies among private institutions. Experiences, perceptions of roles and duties and value judgments can only partly be understood through quantitative methods, and our study should to be supplemented by qualitative in-depth studies and observations studies.

## Conclusion

This is a unique study of the attitudes and practices of a nationally representative sample of Ethiopian physicians’ regarding the protection of patients and their families from health expenses in a low-income country on the path to universal health coverage. Ethiopian physicians view themselves as both gatekeepers of public resources and patient advocates as well as stewards of the family. Their concern for family finances seems to outweigh their concern for the institutional resources, and when forced to choose which adverse outcomes to avoid, their concern for family welfare seems to sometimes outweigh potentially individual health benefit. If further research confirms these findings, the ethical implications of the clinical trade-off between individual health benefit and family welfare warrants further ethical analysis. Given the pivotal role that clinicians play, their experiences and perceptions should be taken into account as policy makers make coverage decisions in order to adequately protect the public from the financial burden of health care.
